# Assessing Wine Grape Cultivar Susceptibility to Spotted Wing Drosophila and Melanogaster-Type Drosophila in Hungarian Vineyards: Effects of Berry Integrity and Insights into Larval Interactions

**DOI:** 10.3390/insects16050497

**Published:** 2025-05-05

**Authors:** Abir Ibn Amor, Ágnes Kukorellyné Szénási, Csaba Németh, Ferenc Deutsch, Balázs Kiss

**Affiliations:** 1 Department of Integrated Plant Protection, Institute of Plant Protection, Hungarian University of Agriculture and Life Sciences, 2100 Gödöllő, Hungary; abirtunisie@gmail.com; 2 Badacsony Research Station, Institute of Viticulture and Oenology, Hungarian University of Agriculture and Life Sciences, 8261 Badacsony, Hungary; 3 Plant Protection Institute, HUN-REN Centre for Agricultural Research, 1116 Budapest, Hungarykiss.balazs@atk.hun-ren.hu (B.K.)

**Keywords:** *Drosophila suzukii* (SWD), infestation dynamics, berry injury, interspecific competition, varietal differences, *Vitis vinifera*, cabernet

## Abstract

The invasive spotted wing Drosophila (SWD) represents new challenges for European and North American fruit producers. The aim of our study was to examine wine grape cultivar susceptibility to this pest and melanogaster-type Drosophila (MTD) by surveying drosophilid populations using field traps and conducting emergence tests. We assessed fly development from intact and artificially injured berries collected from four cultivars. Berries were incubated individually and in pooled samples to evaluate infestation patterns and potential larval interactions. Although grapes are generally considered less favorable hosts for SWD, the pest was consistently present across all vineyard plots. Infestation levels differed significantly among cultivars, with the Hungarian white cultivar Furmint being the most susceptible, while French-origin red cultivars Cabernet Franc and Cabernet Sauvignon, along with the other Hungarian cultivar Rózsakő, were less susceptible. Berry integrity played a crucial role: intact berries showed minimal infestation, whereas physical injuries led to a substantial and significant increase in infestation rates and fly emergence. In contrast to SWD-dominated trap catches and the nearly equal proportions of SWD and MTD observed in intact berries, injured berries were predominantly colonized by MTD. This dominance became even more pronounced in pooled samples, suggesting that larval competition in shared environments favors MTD over SWD. These findings underscore the importance of grape cultivar traits and berry condition in shaping Drosophila infestation dynamics. Further research into the chemical and ecological drivers of host selection and interspecific interactions is warranted to improve vineyard pest management strategies.

## Simple Summary

The spotted wing Drosophila (SWD) is an invasive pest that primarily targets thin-skinned fruits, while grapes are generally considered less susceptible. Our objective was to evaluate the importance of SWD as a primary pest in Hungary, as well as to get insight into its interaction with other melanogaster-type Drosophilas (MTD). Our results showed a consistent dominance of SWD in field traps in the grape ripening period. While intact berries showed a low overall level of infestation, there were significant differences in susceptibility among grape cultivars. Berry injury substantially increased the number of emerging flies and led to a higher proportion of MTD compared to SWD. This shift in species composition was even more pronounced when collected berries were maintained in pooled samples rather than individually isolated. Our findings highlight the role of SWD in the primary infestation of intact grape berries and underscore the importance of considering interspecific interactions when interpreting results based on fly emergence from field-collected fruits.

## 1. Introduction

The spotted wing Drosophila (SWD—*Drosophila suzukii* Matsumura 1931) (Diptera: Drosophilidae) is an invasive fruit pest native to East Asia. It was first detected outside its native range in Hawaii in 1980 before being reported in 2008 in Spain and California, USA, and later in Italy in 2009. Since then, SWD has spread extensively, invading most parts of Europe and North America. Outbreaks of SWD have severely impacted fruit production, and within a decade, it has become a major pest of several soft-skinned fruit species in numerous regions across Europe [1,2,3,4,5,6,7].

A highly polyphagous pest, SWD infests a broad range of wild and cultivated plants. The varied ripening times of its host fruits enable the species to reproduce throughout much of the growing season. Economically significant damage has been reported in soft fruits such as strawberries, blueberries, raspberries, and cherries. While other fruits, including tomatoes, grapes, and pears, may serve as hosts, they generally do not sustain economically significant damage [5].

Studies have shown that the fruit of *Vitis vinifera* is not highly susceptible to SWD, with oviposition rates and developmental success on grapes being lower than on other berries. For example, Pelton et al. [8] found no correlation between adult abundance in traps and larval presence in grapes in a study conducted in Southern Wisconsin vineyards. Similarly, Lee et al. [9] demonstrated that grapes have low susceptibility to SWD. Poyet et al. [10] investigated SWD performance on different fruit substrates and found that nearly half of the tested plant species supported complete SWD development; however, grapes were among the less favorable hosts for larval success.

Contrary to these findings, other studies suggest that grapes can become a suitable host under certain conditions. In European viticulture, significant SWD damage was recorded in Northern Italy in 2012, in Southwest Germany in 2014, and in France from 2014 onwards [11,12,13]. Additional reports from Switzerland indicated that SWD can damage grapes, leading to increased production costs [14,15,16]. The damage caused by SWD in vineyards often extends beyond direct oviposition. Entry holes created during oviposition can result in secondary damage, such as sour rot outbreaks, and facilitate infestation by other Drosophilas [12,17].

In Hungary, SWD was first reported in 2012 and has since rapidly spread throughout the country. Within five years, it caused damage to several fruit crops, including raspberries and blackberries [18,19]. However, reports on the relationship between trap catches and larval damage on different fruits are often inconsistent [7].

Despite the growing importance of SWD in vineyards, relatively little research has explored its interactions with other drosophilids. For instance, few studies have investigated how associations between SWD and other *Drosophila* species influence infestation dynamics [20].

These contradictory findings underscore the need to investigate grape cultivar susceptibility to SWD, with particular attention to interactions with other drosophilids, as well as the parameters influencing infestation rates. The present study aims to evaluate the susceptibility of four wine grape cultivars used in Hungarian vineyards and to distinguish primary and secondary damages due to *Drosophila* in grapes.

## 2. Materials and Methods

### 2.1. Location and Cultivars

The study was conducted at Badacsony Research Station of the Institute of Viticulture and Oenology of the Hungarian University of Agriculture and Life Sciences. The experimental fields were located within the institute’s 18-hectare surface in the middle of the Badacsony wine region. The research involved four wine grape cultivars, each grown in separate vineyard plots.

Cabernet Franc, an early-ripening French red wine grape characterized by high acidity, moderate tannins, and thin skin [21,22], was cultivated on a 0.37 ha plot (46.789260° N, 17.489621° E). Cabernet Sauvignon, also a French red cultivar, known for its late ripening, thick skin, and high tannin content [23,24], was planted on a similarly sized 0.37 ha plot. Furmint, a Hungarian white grape with high acidity, thin skin, and a strong potential for sugar and tartaric acid accumulation, was grown on a 0.21 ha parcel (46.788577° N, 17.495701° E) [25]. Rózsakő, another Hungarian white cultivar with medium acidity, firm skin, and a balanced sugar–acid profile, was cultivated on a 0.83 ha plot (46.789614° N, 17.497108° E), interplanted with Kéknyelű in alternating rows [26]. Intact and artificially injured grape berries were collected across the 2023 and 2024 growing seasons to assess the susceptibility of the cultivars to *Drosophila* infestation.

Insecticidal treatments were applied to the vineyard plots in mid to late July in both years, more than one month before the first berry sampling. Acetamiprid and deltamethrin were used in the conventional plots (Cabernet Franc, Cabernet Sauvignon, and Furmint), while pyrethrin was applied in the organically managed Rózsakő block. All treatments preceded the seasonal arrival of spotted wing Drosophila, and no insecticides were used during the critical sampling window.

### 2.2. Bottle Trap Survey

Drosophilid flies were monitored in the experimental plots using 0.5 L bottle traps containing 0.15 L of apple cider vinegar as an attractant. Although alternative baits (e.g., yeast–sugar mixtures or commercial lures) have been reported to offer higher attractiveness to SWD and related species, apple cider vinegar was selected to maintain consistency with established vineyard monitoring protocols and with previous studies [4,11].

Five traps per cultivar were deployed at the upper trellis level within the vine canopy at the first sampling date and were changed at subsequent sampling dates. The captured drosophilids were preserved in 70% ethanol for later identification and quantification in the laboratory.

### 2.3. Intact Berry Treatment

Intact berries were sampled from each grape cultivar (referring to berries that were healthy and not artificially injured before laboratory examination) at three different dates in both years (7 September, 21 September, and 7 October in 2023; 5 September, 19 September, and 3 October in 2024). For all cultivars and sampling dates, the phenological stage of the grapevines was between BBCH 87 and 89, corresponding to full ripeness and harvest maturity. No major phenological differences were observed among the cultivars, which had all reached harvest maturity by the time of the last sampling date.

Sampling was performed randomly along two vineyard rows per cultivar to ensure representative data collection.

The berry samples from each cultivar were transported to the laboratory in mesh bags. There, 50 berries per cultivar and date were placed individually in small isolator vials (Single Berry Analysis), and in parallel, 250 berries were placed in groups of 25 in larger isolator jars. For pooled samples, we had 10 replicates per cultivar.

The collected berries were incubated under controlled conditions (20–23 °C, 50–70% humidity) for 15 days to monitor adult fly emergence. Flies were identified under a microscope and classified as spotted wing Drosophila (SWD) or melanogaster-type Drosophila (MTD), which included the sibling species *D. melanogaster* and *D. simulans*, which exhibit nearly identical morphology [27].

### 2.4. Hurt Berry Treatment

To evaluate the scale of secondary infection by drosophilids, artificial injuries were made on selected berries during the first and second collection dates by making small cuts on the berries using a blade.

**Figure 1:**
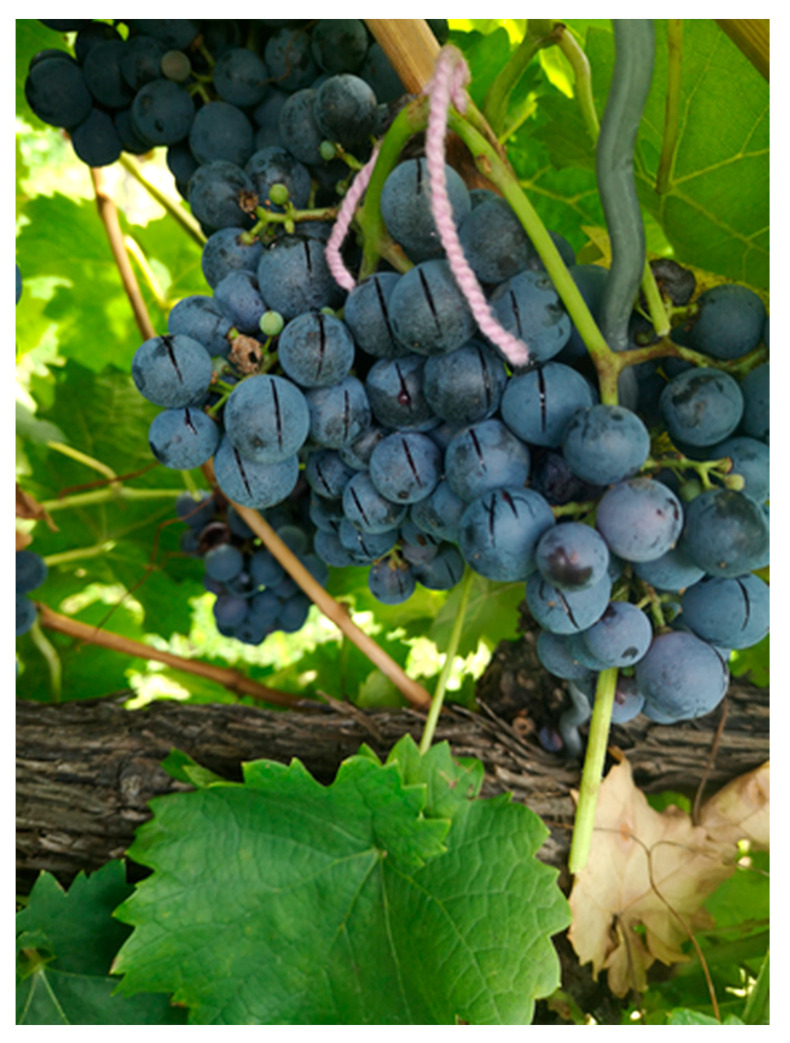
Artificial berry injury in a cluster of Cabernet Franc cultivar.

For each experimental cultivar, five plants were selected in two separate rows, and five clusters were designated for berry collection. Each cluster, consisting of a compact group of berries developing from a single inflorescence, served as a unit for berry injury. Within each designated cluster, several individual berries were manually injured, and the clusters were marked for identification (Figure 1).

Injured berries were sampled and analyzed alongside intact berries on the following collection dates. The samples were processed in the same way as for intact berries: 50 injured berries per cultivar were placed individually in vials (Single Berry Analysis), and 25 injured berries per cultivar were placed in jars (Pooled Sample Analysis; 10 replicates per cultivar). The emergence of adult flies was monitored under the same laboratory conditions (20–23 °C, 50–70% humidity, 15 days) as in the case of the intact berry treatment. Species identification was performed as described above.

### 2.5. Statistical Analysis

All statistical analyses were performed using Python (version 3.11.6). Temporal trends in fly catches across years were analyzed using the Mann–Whitney U test. Differences in trap catches among grape cultivars were evaluated with the Kruskal–Wallis H test, followed by Mann–Whitney U tests with Bonferroni corrections for pairwise comparisons.

The proportions of infested berries across sampling dates were compared using chi-square tests. Varietal differences in infestation rates were assessed using additional Kruskal–Wallis tests, followed by Mann–Whitney U tests. The effect of berry treatment (intact vs. injured) on infestation levels was analyzed using chi-square tests, which were also used to compare the relative proportions of emerging SWD and MTD.

To compare grouping methods, chi-square tests were used to assess differences in species composition between pooled and single berry samples, while Wilcoxon rank sum tests were used to compare total fly counts. In all analyses, *p* < 0.05 was considered statistically significant, with Bonferroni corrections applied when appropriate.

## 3. Results

### 3.1. Bottle Trap Catches

**Figure 2:**
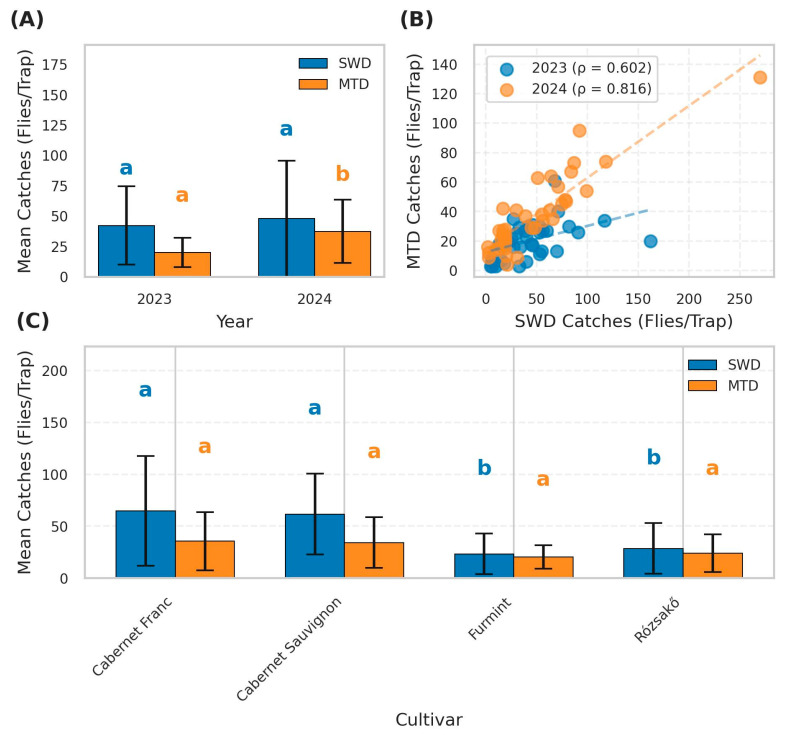
Summary of insect trap catch data comparing spotted wing Drosophila (SWD) and melanogaster-type Drosophila (MTD) across years and cultivars. (**A**) Bar plot comparing mean catches (flies/trap) of SWD and MTD in 2023 and 2024, with error bars representing standard deviation (SD). Different letters above bars indicate statistically significant differences between years for each species (*p* < 0.05). MTD catches differed significantly between years (‘a’ and ‘b’), while SWD catches did not (both marked ‘a’). (**B**) Scatter plot showing the correlation between SWD and MTD catches for 2023 and 2024, with Spearman’s rank correlation coefficients (ρ = 0.602 for 2023; ρ = 0.816 for 2024) and fitted regression lines (dashed). (**C**) Bar plot comparing mean catches (± SD) of SWD and MTD across four grape cultivars (Cabernet Franc, Cabernet Sauvignon, Furmint, and Rózsakő). Different letters above bars indicate statistically significant differences between cultivars for each species separately (*p* < 0.05).

Grapevine monitoring using bottle traps revealed a consistent presence of both SWD and MTD across all sampling dates in 2023 and 2024. All values are reported as mean ± SD. The high standard deviation values likely reflect substantial variation in trap catches between sampling dates within each season. This variation is attributable to the population dynamics of SWD, particularly its rapid increase during early autumn, when immigration into vineyards typically intensifies. Although SWD catches had a slightly higher mean in 2024 (47.85 ± 47.81 flies per trap) compared to 2023 (42.26 ± 32.18 flies per trap), the difference was not statistically significant (Mann–Whitney U test, *p* = 0.849). In contrast, MTD densities increased substantially from 20.00 ± 12.14 flies per trap in 2023 to 37.38 ± 26.19 flies per trap in 2024, representing a statistically significant difference (Mann–Whitney U test, *p* < 0.001) (Figure 2A).

A strong positive correlation was observed between SWD and MTD populations overall (Spearman’s ρ = 0.686, *p* < 0.001), with the strength of the correlation varying between years (ρ = 0.602, *p* < 0.001 in 2023 and ρ = 0.816, *p* < 0.001 in 2024). The maximum SWD catch (270 flies per trap) was recorded on September 19, 2024, which corresponded to the late ripening stage of the grape cultivars, approximately two weeks before harvest, confirming its numerical dominance in certain periods of the season. Its relative share within total Drosophila catches declined from 67.87% in 2023 to 56.14% in 2024, suggesting a slight shift in pest composition (Figure 2B).

Within-year comparisons revealed significant seasonal increases in both pest populations, coinciding with berry development between advanced ripening stages. In 2023, SWD densities rose from 30.95 ± 24.67 flies per trap on September 7 (BBCH 87: majority of berries having reached typical size and color) to 54.16 ± 35.40 flies per trap on October 7 (BBCH 89: full ripeness, harvest-ready) (t = −2.386, *p* = 0.022, Cohen’s d = 0.764). MTD densities increased from 15.95 ± 9.09 to 24.26 ± 13.66 flies per trap over the same period (t = −2.248, *p* = 0.031, d = 0.720). In 2024, similar patterns were observed, with SWD densities increasing from 30.00 ± 22.06 flies per trap on September 5 (BBCH 87) to 64.80 ± 59.08 flies per trap on October 3 (BBCH 89) (t = −2.411, *p* = 0.021, d = 0.773). MTD densities rose from 25.00 ± 14.70 to 49.15 ± 29.46 flies per trap during the same phenological transition (t = −3.212, *p* = 0.003, d = 1.029).

Significant differences were observed in SWD catches by cultivar (Kruskal–Wallis *H* = 24.94, *p* < 0.0001), whereas MTD catches did not differ significantly (*H* = 5.34, *p* = 0.15). Significantly higher catches of SWD were recorded in the red wine grape cultivars than in the white ones. The overall SWD counts (mean ± SD) were 64.8 ± 52.8 for Cabernet Franc, 61.6 ± 38.9 for Cabernet Sauvignon, 23.3 ± 19.5 for Furmint, and 28.5 ± 24.4 for Rózsakő. Pairwise comparisons showed significant differences between red and white cultivars, with no significant differences between Cabernet Franc and Cabernet Sauvignon or between Rózsakő and Furmint (Figure 2C).

### 3.2. Fly Emergence from Unhurt Single Berries

#### 3.2.1. Proportion of Infested Berries by Sampling Dates

**Figure 3:**
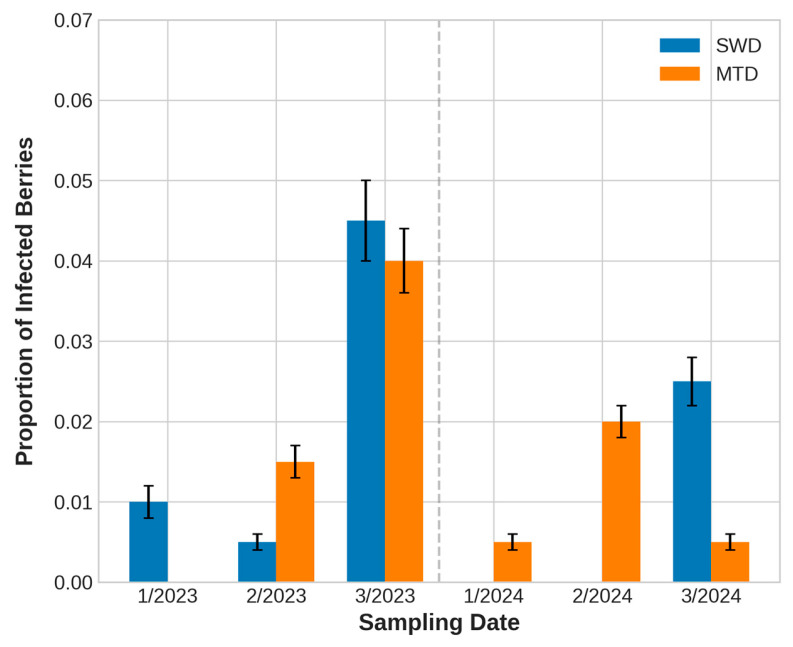
Temporal dynamics of spotted wing Drosophila (SWD) and melanogaster-type Drosophila (MTD) infestation rates in intact berries across sampling dates in 2023 and 2024. Error bars show 95% confidence intervals. The dashed vertical line separates observations between years.

Overall, drosophilid emergence was observed in 33 out of 1200 berries examined (≈2.8%). Of these infested berries, 16 contained only SWD, 16 only MTD, and one contained both species. When infestation patterns were analyzed across sampling dates, notable year-to-year differences emerged (Figure 3).

In 2023, the proportion of SWD-infected berries increased from 1.0% noted on the first sampling date to 4.5% on the third, while MTD infestation rose from 0% to 1.5%, peaking at 4.0% on the third sampling date (χ^2^ = 9.69, *p* = 0.0079 for SWD; χ^2^ = 9.08, *p* = 0.0107 for MTD) (Figure 3).

In 2024, SWD emerged only from the berries collected on the third date, while the MTD emergence rate was relatively stable on the three sampling dates (0.5%, 2.0%, and 0.5%) (Figure 3).

#### 3.2.2. Varietal Differences in Infestation Rates

Significant differences were found among the cultivars in the infestation rates of SWD and MTD. For SWD, Furmint consistently showed the highest infestation rates, with 4.7% in 2023 and 4.0% in 2024, while Rózsakő showed no infestation (0%) in both years (Table 1). In 2023, although the overall effect of the cultivar was not statistically significant (Kruskal–Wallis *H* = 4.61, *p* = 0.2029), Furmint’s infestation rates were notably higher than those of Rózsakő (4.7% vs. 0.0%). In 2024, the effect of cultivar was significant (*H* = 18.92, *p* < 0.001), Furmint being significantly more infected by SWD than the other cultivars in pairwise comparisons (all *p* < 0.01) (Table 1).

For MTD, Furmint again had the highest infestation rates, with 7.3% in 2023 and 4.7% in 2024. These were substantially higher than those in Cabernet Franc (0.7% and 0.0%) and Cabernet Sauvignon (0.0% in both years), while Rózsakő consistently recorded the lowest MTD infestation (1.3% in 2023 and 0.0% in 2024). The overall effect of cultivar in MTD infestation was highly significant in both 2023 (Kruskal–Wallis *H* = 25.26, *p* < 0.0001) and 2024 (*H* = 22.15, *p* < 0.0001). Mann–Whitney U tests confirmed that Furmint had significantly higher infestation rates than Rózsakő in both years (*p* < 0.01) (Table 1).

Despite inter-annual variation, Furmint consistently emerged as the most susceptible cultivar to both MTD and SWD, while Rózsakő consistently showed the lowest susceptibility.

### 3.3. Fly Emergence from Unhurt Pooled Berries (Varietal Differences)

**Figure 4:**
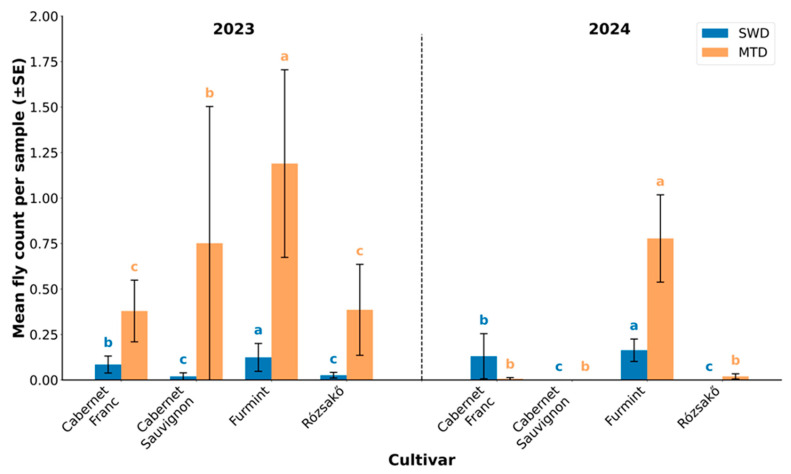
Mean fly count per sample for spotted wing Drosophila (SWD) and melanogaster-type Drosophila (MTD) from unhurt pooled berries across different grape cultivars in 2023 and 2024. Different letters above bars indicate statistically significant differences among cultivars within each year and species (*p* < 0.05). Bars sharing the same letter are not significantly different. Error bars represent the standard error of the mean (±SE).

Consistent with the infection rates observed in single-berry samples, significant differences were found in the number of flies emerging from pooled samples among grape cultivars. For SWD, significant differences were detected among cultivars (Kruskal–Wallis test, *p* < 0.05 for both 2023 and 2024). The highest SWD emergence was recorded in Furmint berries (*n* = 26 in 2023; *n* = 31 in 2024), followed by Cabernet Franc (*n* = 15 in 2023; *n* = 20 in 2024). In contrast, Rózsakő and Cabernet Sauvignon consistently exhibited low SWD emergence (*n* = 4 and 0 in 2023 and 2024, respectively, for Rózsakő; *n* = 8 and 0 for Cabernet Sauvignon) (Figure 4).

For MTD, significant differences were also observed (*p* < 0.05 in both years). Furmint again showed the highest emergence (*n* = 193 in 2023; *n* = 126 in 2024), followed by Cabernet Sauvignon (*n* = 115 in 2023; *n* = 0 in 2024). Rózsakő and Cabernet Franc exhibited moderate and comparable levels, with *n* = 61 and 3 in 2023 and 2024, respectively, for Rózsakő, and *n* = 59 and 1 for Cabernet Franc. These findings reinforce that Furmint is the most susceptible cultivar to both pests, while Rózsakő appears to be the least susceptible (Figure 4).

### 3.4. Comparison of Infestation of Intact and Hurt Single Berries

Our results have shown low susceptibility of intact berries across both years, maintaining the rate of uninfected berries above 96%. Hurting berries significantly increased berry vulnerability to pest colonization, with hurt berries showing markedly higher infestation rates (χ^2^ = 3341.34, df = 3, *p* < 0.001). In 2023, 46% of hurt berries were infested, predominantly by MTD (32.25%), while 12% showed combined SWD + MTD presence. In 2024, infestation in injured berries dropped to 22%, with MTD accounting for 16%, SWD for 5.25%, and only 0.75% showing mixed-species infestation. Notably, SWD infestation was slightly higher in 2024 than in the previous year (Table 2).

### 3.5. Differences in Drosophila Composition in Isolated and Pooled Samples

The emergence ratios of SWD and MTD varied considerably according to berry condition (intact vs. injured) and berry keeping method (isolated vs. pooled). These variations were highly consistent in the two experimental years. For individually treated intact berries, the emergence of SWD and MTD adult flies was nearly equal (approximately 50% each year). By contrast, injured berries consistently showed higher MTD emergence, with MTD representing over 84% of the emergent flies regardless of whether berries were treated individually or in pooled groups (χ^2^ test, *p* < 0.001; Table 3).

Moreover, when intact berries were treated as pooled samples, thus allowing interactions among larvae developing in different berries, the proportion of the emerging SWD flies was significantly lower. Specifically, the ratio of SWD to MTD in intact pooled berries was only 8.6% in 2023 (χ^2^ test, *p* < 0.001) and approximately 26.8% in 2024. Our results emphasize that both berry injury and pooled treatment led to species interactions in favor of MTD (Table 3).

The total number of emerged flies showed marked differences between single and pooled berry conditions, with pooled berries consistently producing higher counts than single berries in both intact and injured conditions. This discrepancy might be due to resource limitations for larvae in single berries (Table 3).

## 4. Discussion

Although grapes are considered less favorable hosts, our results show that SWD adults were consistently present in the vineyards throughout all monitoring periods, corroborating the numerous reports on the trappability of SWD in grape plantations [11,27,28]. In autumn, SWD and MTD become part of the aeroplankton, even in places without fruit availability, where they are found in high numbers due to microclimatic effects [28,29,30]. This widespread presence may also be supported by off-season and off-crop refuges in the surrounding landscape, as discussed by Aguiar et al. [31]. This suggests that their presence does not necessarily indicate an infestation, as individuals may disperse widely without ovipositing. Similar effects have been observed in cherry orchards, highlighting how habitat structure influences drosophilid dispersal and persistence. Thus, the presence of SWD does not necessarily mean it actively infests grapes, as factors like dispersal behavior, habitat suitability, and competition influence actual infestation rates.

In other studies, in Italy, France, Turkey, and Brazil, SWD has been associated with considerable damage in grapes. SWD caused significant infestations in Northern Italy in 2012, in Southwest Germany in 2014, and in France since 2014 [11,12,13]. However, in Hungary, the situation of SWD in vineyards remains less studied [32]. Previous research has primarily focused on SWD as an invasive pest without considering the broader composition of Drosophila species in vineyards [9,28]. Our study evaluates the susceptibility of four wine grape cultivars produced in Hungary to both SWD and MTD, acknowledging the possible complexity of drosophilid fauna in vineyards.

The higher catches of SWD observed in red grape cultivars compared to white cultivars suggest a potential link between host preference and olfactory cues. Red wine has been shown to act as a lure for Drosophila species, particularly *D. melanogaster*, due to its fermentation byproducts and volatile organic compounds. Given that red grape cultivars have higher anthocyanin and phenolic content, these compounds may play a role in attracting SWD for oviposition. This aligns with findings that SWD shows greater attraction to fruits with increased sugar and volatile emissions, potentially explaining its higher densities in red cultivars [33,34,35]. It should be noted that the performance of baited traps may be influenced by various environmental and physiological factors, including the seasonal conditions and host-searching behavior of the flies [36,37,38].

The overall proportion of intact berries (non-artificially injured) containing Drosophila individuals was relatively low, but in some cases, not negligible as primary damage, especially when considering the effect of larval activity on wine quality. Studies have shown that even low levels of drosophilid larval activity can influence grape fermentation, leading to changes in yeast composition, increased acetic acid levels, and potential spoilage of the final wine product [11,39]. Although infestation levels in intact berries were relatively low, the consistent presence of SWD in traps and its ability to exploit damaged fruit suggest that its potential impact should not be overlooked. This study is not only intended to assess current infestation levels but also to explore the ecological factors and cultivar-specific traits that may influence susceptibility, as shown by our results, where the overall mean conceals the much higher infestation rates observed in certain individual cases, especially at later sampling dates, even under standard vineyard conditions. While the current data do not indicate widespread damage, understanding these interactions is essential for anticipating future risks, particularly in seasons with increased berry injury, delayed harvest, or shifts in pest behavior. Documenting such dynamics provides a valuable foundation for early detection, informed monitoring, and preventative management strategies.

Differences in infection rates between cultivars may be attributed to factors such as skin thickness, acidity, and sugar content, which can influence oviposition preference and larval survival [40,41,42]. With emergent flies from single berries and pooled samples, we found that Furmint was the most susceptible cultivar, likely due to its tendency for berry splitting, skin thinning during ripening, and late maturation, which aligns with peak SWD activity [43,44,45]. Cabernet Sauvignon and Cabernet Franc also showed high susceptibility, with low skin elasticity facilitating oviposition [8,9,44]. In contrast, Rózsakő was the least susceptible to *Drosophila suzukii* infestation, likely due to its firm berry skin acting as a physical barrier. Created in 1957 by Ferenc Király as a cross between Kéknyelű and Budai Zöld, Rózsakő may have inherited this trait from its parent cultivar, Kéknyelű. Throughout the study, Rózsakő berries remained noticeably firmer than the other tested cultivars, which may explain their lower infestation rates [45].

It is surprising that the dominance of SWD in trap catches was not reflected in the ratio of infected berries or emerged flies. Even in the case of intact berries, where the adaptive ovipositor of SWD should provide a competitive advantage over MTD in laying eggs, the proportion of emerged MTD flies was higher than in the bottle traps. This discrepancy suggests that factors such as larval competition, microbial interactions, and post-oviposition mortality may play a key role in shaping species composition within infested fruit [46,47,48]. Additionally, it is possible that although SWD adults were present in high numbers during late ripening, actual oviposition into intact berries was limited by subtle differences in skin firmness, fruit chemistry, or SWD behavioral preferences at that stage. As Leach et al. [49] point out, fruit phenology and environmental conditions can strongly influence the success of oviposition and larval development, which may explain why trap captures did not translate into proportionally higher emergence from berries in our study.

Not surprisingly, artificial injury significantly increased the probability of Drosophila infestation, with a much stronger effect on MTD than on SWD. The damage to berry integrity provided an entry point for oviposition, making the fruit more attractive to MTD, which typically prefers decaying or fermenting substrates. SWD, despite having a specialized ovipositor allowing it to infest intact fruit, showed relatively lower infestation rates compared to MTD in injured berries [50,51,52].

Our findings confirm that SWD infestation rates were significantly higher in injured berries, supporting previous research indicating that host susceptibility to SWD increases when table grapes are damaged. Injured fruits provide a more favorable environment for SWD, facilitating easier oviposition and offering increased nutritional resources, which can lead to population growth over time [53]. Before injury, the intact berry skin acted as a physical barrier, particularly limiting MTD oviposition. Once berries were wounded, oviposition sites became accessible and fermentation accelerated, making the berries more attractive to *D. melanogaster* and *D. simulans*. The preference of these species for overripe, damaged fruit likely contributes to this outcome [54].

Furthermore, when berries were pooled (placing multiple intact fruits in close proximity and allowing potential interactions between larvae), MTD emergence became even more dominant. Although the berries were not visibly damaged, their arrangement in groups may have allowed larvae to encounter one another more easily or be exposed to shared chemical or spatial cues, similar to what may occur in natural fruit clusters. Such proximity can influence developmental outcomes, potentially enhancing competitive advantages for MTD. These effects may also include indirect forms of interference or even cannibalism under crowded or deteriorating post-harvest conditions. Pooled conditions likely provide greater resources and promote competition among larvae, favoring MTD development over SWD.

This unexpected result highlights that rearing Drosophila from fruit may not necessarily provide a reliable representation of the species composition of primary pests responsible for the initial damage. These findings emphasize that both berry injury and pooling contribute to species interactions that favor MTD over SWD, highlighting the importance of preventing berry damage in pest management strategies.

## 5. Conclusions

Our findings demonstrate that wine grape cultivars vary significantly in their susceptibility to *Drosophila suzukii* (SWD) and melanogaster-type Drosophila (MTD), with Furmint being the most vulnerable and Rózsakő the least susceptible. These cultivar-specific differences highlight the potential of using low-susceptibility grape types as a tool in integrated vineyard pest management.

The study also reveals the critical role of berry integrity in infestation dynamics. Once injured, berries became substantially more vulnerable to colonization, especially by MTD, which consistently outcompeted SWD in damaged conditions and pooled environments. Even among intact berries, pooling appeared to influence larval emergence, likely due to post-harvest microenvironmental changes such as increased humidity, microbial activity, or subtle skin weakening, which may have allowed limited larval movement or indirect species interactions. This suggests that fruit condition and larval interactions are key determinants of infestation outcomes.

While SWD dominated trap catches, MTD emerged more frequently from fruit, indicating that emergence data may better reflect actual infestation dynamics.

Together, these results emphasize that maintaining berry integrity, selecting less susceptible cultivars, and understanding interspecific dynamics are essential strategies for sustainable pest control in vineyards.

Future research should investigate the chemical and mechanical properties that confer lower susceptibility and further explore larval interactions as drivers of species success within infested fruit. These insights will contribute to more targeted and environmentally conscious pest management approaches.

## Figures and Tables

**Table 1:** Proportions of single berries containing spotted wing Drosophila (SWD) and melanogaster-type Drosophila (MTD) individuals in 2023 and 2024 (Total N = 300 for each cultivar).

Cultivar	SWD 2023 (%)	SWD 2024 (%)	MTD 2023 (%)	MTD 2024 (%)
Cabernet Franc	1.3	0.0	0.7	0.0
Cabernet Sauvignon	3.3	0.0	0.0	0.0
Furmint	4.7	4.0	7.3	4.7
Rózsakő	0.0	0.0	1.3	0.0

**Table 2:** Percentage distribution of spotted wing Drosophila (SWD) and melanogaster-type Drosophila (MTD) infestations in intact and hurt single berries during the 2023–2024 monitoring period.

Year	Condition	Non- Infested (%)	MTD (%)	SWD (%)	SWD + MTD (%)	Total (N)
2023	Intact	96.33	1.67	1.83	0.17	600
2023	Hurt	54.0	32.25	1.75	12.0	400
2024	Intact	98.17	1.0	0.83	0	600
2024	Hurt	78.0	16.0	5.25	0.75	400

**Table 3:** Proportional emergence of spotted wing Drosophila (SWD) and melanogaster-type Drosophila (MTD) across different conditions and years (N = total number of emergent flies).

Year	Condition	MTD (%)	SWD (%)	Total (N)
2023	Intact Single Berry	50.0	50.0	28
	Intact Pooled Berries	91.4	8.6	453
	Injured Single Berry	90.5	9.5	1037
	Injured Pooled Berries	97.0	3.0	2074
2024	Intact Single Berry	53.8	46.2	13
	Intact Pooled Berries	73.2	26.8	168
	Injured Single Berry	84.8	15.2	243
	Injured Pooled Berries	90.3	9.7	1813

## Data Availability

Data are contained within the article.
